# Toll-like receptor 2 drives liver senescence and fibrosis in aging through gut-derived microbial signaling

**DOI:** 10.1186/s11658-026-00881-4

**Published:** 2026-03-03

**Authors:** Annette Brandt, Raphaela Staltner, Anja Baumann, Katharina Burger, Julia Jelleschitz, Patricia Oliveira Prada, Annika Höhn, Florian Kopp, Jordi Mayneris-Perxachs, José Manuel Fernández-Real, Ina Bergheim

**Affiliations:** 1 Department of Nutritional Sciences, Molecular Nutritional Science, University of Vienna, Vienna, Austria; 2 German Institute of Human Nutrition Potsdam-Rehbrücke (DIfE), Nuthetal, Germany; 3 German Center for Diabetes Research (DZD), Munich-Neuherberg, Germany; 4 Clinical Pharmacy Group, Department of Pharmaceutical Sciences, University of Vienna, Vienna, Austria; 5 Integrative Systems Medicine and Biology Group, Girona Biomedical Research Institute (IDIBGI-CERCA), Salt, Spain; 6 CIBER Fisiopatología de La Obesidad y Nutrición (CIBERobn), Madrid, Spain; 7 Department of Diabetes, Endocrinology and Nutrition, Institut d’Investigació Biomèdica de Girona (IDIBGI), Girona, Spain; 8 CIBERobn Fisiopatología de la Obesidad y Nutrición; and Department of Medical Sciences, School of Medicine, University of Girona, Girona, Spain

**Keywords:** Aging, Lipoteichoic acid, Hepatic inflammation, Fibrosis, TLR2, Senescence

## Abstract

**Background:**

While the role of endotoxins from Gram-negative bacteria has been studied extensively, the contribution of Gram-positive bacterial components—particularly those activating toll-like receptor 2 (TLR2), such as lipoteichoic acid (LTA)—to liver aging, inflammation, and fibrosis remains poorly understood. Here, we investigated the role of TLR2 and its ligand LTA in liver aging by using murine models, in vitro experiments, and human samples from young and elderly individuals.

**Methods:**

TLR2 ligands were evaluated in serum samples from young (aged 21–33 years) and elderly (aged 65–77 years) healthy individuals. Markers of liver damage, senescence, and inflammation were assessed in 4- and 20-month-old male C57BL/6 and TLR2 knockout (TLR2^−/−^) mice. In addition, 17-month-old male C57BL/6 mice were treated either with the TLR2 inhibitor ortho-vanillin (60 mg/kg BW in drinking water) or plain water for 4 months and markers as determined above were assessed. The presence of markers of senescence was measured in J774A.1 cells and human peripheral blood mononuclear cells stimulated with LTA.

**Results:**

In humans and mice, aging was associated with significantly elevated circulating levels of TLR2 ligands. In aging mice, this was accompanied by increased hepatic *Tlr2* mRNA expression. Strikingly, 20-month-old male TLR2^−/−^ mice exhibited reduced markers of senescence (e.g., plasma plasminogen activator inhibitor-1 and liver *p16* expression), inflammation (e.g., hepatic neutrophil infiltration, *Il1b* mRNA expression), and fibrosis (e.g., *α-smooth muscle actin* expression, Sirius Red staining), compared with age-matched wild-type controls. Similarly, in aged male C57BL/6 J mice showing first signs of impaired intestinal barrier function i.e., rising peripheral TLR2 ligand levels in plasma, treatment with the TLR2 inhibitor ortho-vanillin for 4 months attenuated the progression of liver aging as indicated by attenuated senescence, liver inflammation, and fibrosis. In vitro, stimulation of J774A.1 macrophages and human peripheral blood mononuclear cells with LTA induced the expression of senescence-associated genes *p16* and *p21*.

**Conclusions:**

Collectively, these findings suggest that increased translocation of TLR2 ligands and subsequent activation of TLR2-dependent pathways play a critical role in age-associated liver degeneration. Targeting TLR2 signaling may therefore represent a promising therapeutic approach to mitigate hepatic aging and associated pathologies.

**Graphical Abstract:**

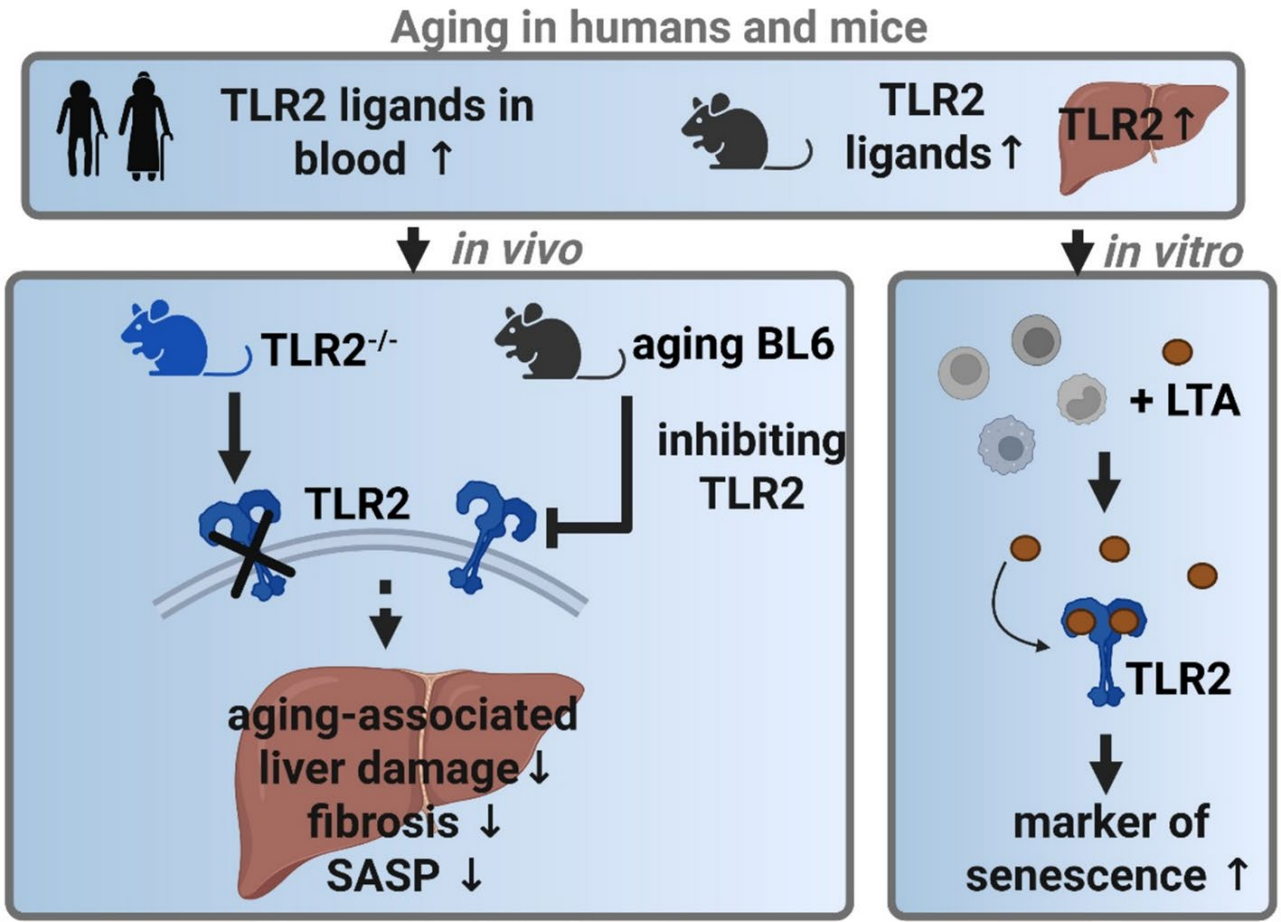

**Supplementary Information:**

The online version contains supplementary material available at 10.1186/s11658-026-00881-4.

## Background

While globally the overall life expectancy is still increasing, healthy lifespan is not increasing at the same rate [1]. Indeed, it has been shown that in the European Union, life expectancy was 77.9 years in males and 83.3 years in females in 2022; however, epidemiological data further suggest that only ~80% (62.4 years) and ~75% (62.8 years), respectively, were on average spent healthy [2, 3]. Old age has been identified as an independent risk factor for the development of various chronic inflammatory diseases, including type 2 diabetes, dementia, and liver diseases [4, 5].

The liver was once considered largely immune to senescence owing to its remarkable capacity for proliferation and regeneration [6]. However, advances in the understanding of organismal aging have revealed that the liver is also subject to age-related changes. These include increased intrahepatic accumulation of lipofuscin, elevated collagen fiber content, and a higher number of senescent hepatocytes [7]. In addition, aging is associated with reduced hepatic blood flow, diminished proliferative and regenerative capacity, and a decline in anabolic functions [8]. Cross-species studies have also demonstrated that hepatic inflammation—and even fibrosis—can occur with advancing age, even in the absence of common triggers such as viral infections, alcohol consumption, overweight, or insulin resistance [9–11]. Despite extensive research, the mechanisms underlying this age-related decline in liver function remain incompletely understood. Studies suggest that similar to some steatotic liver diseases such as alcohol-associated liver disease and metabolic dysfunction-associated steatotic liver disease (MASLD) aging-related liver decline may be linked through the so called “gut–liver–axis” to alterations of intestinal microbiota and barrier dysfunction [9, 12, 13]. Studies also indicate that liver damage is often accompanied by altered bile acid metabolism, which also has been linked to alterations of intestinal barrier function [13].

Supporting the hypothesis that changes of the “gut–liver–axis” are critical in aging-related liver decline, recent studies found that even healthy aging is associated with alterations of intestinal microbiota composition, alterations of bile acid metabolism, and impairments of intestinal barrier function, as well as an increased translocation of bacterial toxins [9, 12, 14–16]. The latter has been shown to result in an induction of pattern recognition receptors (PRR) such as toll-like receptors (TLRs) and associated proinflammatory signaling cascades in liver tissue [9, 10, 17]. These proinflammatory signaling cascades might drive senescence and add to the so called “inflammaging,” a state of low-grade inflammation resulting in cell and organ damage [18]. It was previously shown by us that cluster of differentiation 14 (CD14) and lipopolysaccharide binding protein (LBP) protein levels are higher in healthy elderly humans when compared with healthy young individuals. These increases are associated with elevated levels of TLR2 and TLR4 ligands (e.g., lipoteichoic acid (LTA), peptidoglycan and endotoxin) [12]. Whether an activation of TLR2 and its signaling cascades is also critical in age-associated liver decline remains unclear. Indeed, TLR2, forming homodimers (TLR2/2) and heterodimers with TLR1 (TLR2/1) or TLR6 (TLR2/6) and in rare cases also with TLR10 (TLR2/10), recognizing pathogen-associated molecular patterns (PAMPs) from Gram-positive bacteria seems to exhibit both pro- and antiinflammatory capacities (for an overview see [19]). Nonetheless, TLR2 ligands such as LTA have been implicated in promoting hepatic inflammation and fibrosis, and have also been associated with the induction of cellular senescence [20–23].

Building on this background, the present study aimed to further elucidate the role of TLR2 in the development of senescence and age-related liver decline, using both mouse models and human samples.

## Methods

### Human subjects

Fasting serum samples from 13 young, healthy, participants (aged 21–33 years) collected in the due course of baseline visits for nutritional intervention studies in our department at University of Vienna, Austria (Details on ethics and consent please see Declaration section at the end of the manuscript), and from 12 healthy elderly subjects (aged 65–77 years) from the Aging Imageomics Study [24] were collected. The latter is an observational study including participants residing in the province of Girona (Northeast Catalonia, Spain). Detailed information have been published before [25], and details on ethics and consent are provided below. None of the subjects included in this analysis had any of the following conditions: metabolic diseases such as cardiovascular diseases, type 2 diabetes, or metabolic dysfunction-associated steatotic liver disease; chronic inflammatory diseases; malignant diseases; or obesity (defined as a BMI > 20.0 but < 25.0 kg/m^2^).

### Mice aging experiments

All animals were handled in accordance with the European Convention for the Protection of Vertebrate Animals used for Experimental and Other Scientific Purposes and housed in a specific-pathogen-free barrier facility accredited by the Association for Assessment and Accreditation of Laboratory Animal Care. All experiments were approved by the local institutional animal care and use committee (Details on ethics and consent please see Declaration section at the end of the manuscript.). Experiment 1: Blood and tissue were collected from 4-, 16-, and 20-month (mo)-old male C57BL/6J mice as detailed below. Experiment 2: In a second experiment male C57BL/6J (BL6, #000664, The Jackson Laboratory, USA) and TLR2 Knockout (TLR2^−/−^, B6.129-Tlr2tm1Kir/J, #004650, The Jackson Laboratory, USA) mice, bred and housed in the SPF animal facility at University of Vienna in groups under controlled conditions and having free access to tap water and standard pellet food at all times were aged to 4 (young) and 20 months (old) (*n* = 8–10). As no differences were observed between young C57BL/6J mice and young TLR2^−/−^ mice with respect of signs of senescence or liver damage, young C57BL/6J mice were used as representative young controls. In a third experiment (Experiment 3) 17-month-old male C57BL/6J mice, showing signs of impaired intestinal barrier function as determined by assessing TLR2 ligand concentration in peripheral blood, were either treated with the TLR2 inhibitor ortho-vanillin (60 mg/kg BW in drinking water, Sigma-Aldrich GmbH, Germany, *n* = 8) or plain water for 4 months (*n* = 8). Young (4 months old, *n* = 6) C57BL/6J mice were used as controls. At the end of the experiments, mice were anesthetized with ketamine/xylazine (100 mg ketamine/kg BW; 16 mg xylazine/kg BW, i.p. injection). Blood was collected from the portal vein and the retrobulbar plexus. Liver samples were collected and snap-frozen or fixed in neutral-buffered formalin. In addition, at the end of experiment 3, peritoneal cavity cells (PCCs) were isolated from mice (see below). Sacrifice and all following measurements were performed in a randomized group order.

### Mice feeding experiment

In a pilot experiment, female 8–10 weeks old C57BL/6J and TLR2^−/−^ mice (*n* = 5) were adapted to a liquid diet for 2 weeks and were pair-fed either a liquid control diet (C-diet, 69E% carbohydrates, 12E% fat, 19E% protein; Ssniff, Germany) or a fat-, fructose-, and cholesterol-rich diet (FFC-diet, 60E% carbohydrates, 25E% fat, 15E% protein with 50% wt/wt fructose, and 0.16% wt/wt cholesterol; Ssniff, Germany) for 6 weeks. For pair-feeding, caloric intake was adjusted daily to the group with the lowest caloric intake as detailed before [26]. For diet composition see Supplementary Table 1.

**Table 1:** Baseline characteristics of young and elderly healthy subjects

	Young subjects	Elderly subjects
Male/female	6/7	6/6
Age (years)	26.1 ± 1.1	71.4 ± 1.2*
BMI (kg/m^2)^	22.5 ± 0.5	23.0 ± 0.4
ALT (U/L)	20.4 ± 3.0	20.7 ± 1.8
AST (U/L)	23.2 ± 1.9	24.7 ± 2.2

Data presented as mean ± SEM

*BMI* body mass index, *ALT* alanine aminotransferase, *AST* aspartate aminotransferase

*p < 0.05. *p*-value determined by unpaired t-test (age and BMI) and Mann–Whitney *U* test (ALT and AST)

### Isolation and treatment of peritoneal cavity cells

PCCs being a mix of macrophages, T-, and B-cells [27] were collected as described previously [27]. In brief, 5 mL of ice-cold PBS/3% fetal bovine serum (FBS, PAN Biotech, Germany) was added to the abdominal cavity and the resulting cell suspension was collected and resuspended in RPMI 1640 media (supplemented with 10% FBS). Cells were transferred to cell culture plates. After incubating cells at 37 °C /5% CO_2_ for 2 h, cells were treated with ± 10 µg/ml LTA (from *Staphylococcus aureus*, Sigma-Aldrich GmbH, Germany), and the cell culture media was collected after 2 h and 6 h.

### Treatment of J774A.1 cells

J774A.1 cells (ACC 170, DMSZ, Braunschweig, Germany) were cultured in Dulbecco’s Modified Eagle Medium (Pan Biotech, Germany) supplemented with 10% FBS (PAN Biotech, Germany) and 1% penicillin and streptomycin at 37 °C in a humidified 5% CO_2_ atmosphere. At 80% confluency, cells were challenged with ± 10 µg/ml LTA (from *Staphylococcus aureus*; Sigma-Aldrich GmbH, Germany) for 6 h. Cells were collected in Trizol (TRItidy G™, AppliChem, Germany).

### Isolation and stimulation of human peripheral blood mononuclear cells (PBMC)

PBMCs were isolated from whole blood samples of young, healthy participants in accordance with the ethics committee of the University of Vienna as described before [28] and stimulated with 10 µg/ml LTA for 48 h. Subsequently, cells were lysed in Trizol (TRItidy G™, AppliChem, Germany) and RNA was isolated.

### Everted gut sac model (*ex vivo*)

During tissue collection small intestinal tissue from TLR2^−/−^ mice and C57BL6/J mice from experiment 2 (see above) was rinsed and everted to build everted gut sacs as detailed before [29, 30]. In brief, intestinal permeability was assessed by everting the segments of small intestine, filling them with 1 × Krebs–Henseleit–bicarbonate buffer containing 0.2% bovine serum albumin (KRH), and incubating the sacs for 5 min in 0.1% xylose prepared in KRH. Following incubation, the luminal contents were collected to quantify xylose permeation.

### Histological evaluation of liver sections

Liver sections (4 µm) from paraffin-embedded tissue were stained with hematoxylin and eosin (Sigma-Aldrich GmbH, Germany). Representative pictures were captured at a 100× (insets show selected areas at 630×) magnification using a camera (Leica, Germany) incorporated in a microscope (Leica DM6 B, Leica, Germany). NAFLD Activity Score was used to assess liver inflammation as described previously [31]. Naphthol AS-D Chloroacetate (Specific Esterase) kit (Sigma-Aldrich GmbH, Germany) was used to stain and count neutrophil granulocytes in liver sections. Picrosirius red (Sigma-Aldrich GmbH, Germany) staining was used to determine collagen deposition in liver sections as described previously [9]. Pictures were taken with a camera (DMC 4500, Leica, Germany) incorporated in the microscope (DM6 B, Leica, Germany). Staining was quantified densitometrically using an image-analysis software (Leica Application Suite Version 4.5.0, Leica, Germany) and expressed as the percentage of the microscopic field displaying the default color in randomly selected regions. Large blood vessels were excluded from the analysis. Frozen liver sections (10 µm) were stained for senescence-associated β-galactosidase (SA β-Gal) as detailed by others [32].

### Assessment of TLR1, 2, and 6 ligands and ELISA

TLR1, 2, and 6 ligands in serum of humans and TLR2 ligands in plasma from *vena fascialis* and portal vein of mice were measured using a commercially available HEK293 cell assay transfected with secreted embryonic alkaline phosphatase (SEAP) reporter and murine or human TLRs (Invivogen, France), as detailed previously [33]. The concentration of interleukin 6 (IL6) protein in the cell culture supernatant of LTA-stimulated PCCs and plasminogen-activator inhibitor-1 (PAI-1) concentration in plasma of mice were assessed using commercially available ELISA Kits (mouse IL6 and PAI-1 DuoSet ELISA, Biotechne, USA).

### Hepatic lipid accumulation and blood parameters of liver damage

Triglycerides were extracted from liver tissue and measured as previously described [34]. Aspartate aminotransferase activity (AST) in plasma was measured in experiments 2 and 3 with measurements being carried out in a routine laboratory (Veterinary Medical University of Vienna, Austria).

### RNA isolation and real-time RT-PCR

After extracting RNA from liver tissue and cells using Trizol (TRItidy G™, AppliChem, Germany), a reverse transcription system obtained from Promega GmbH, USA, was used to synthesize cDNA. Real-time polymerase chain reaction (PCR) was performed as described previously [14] to amplify genes listed in Supplementary Table 2, normalized to *18S.*

**Table 2:** Body weight and markers of liver damage as well as markers of intestinal barrier function in 4-month and 20-month-old BL6 and TLR2^−/−^ mice

Parameter	Groups		
	4 months	20 months	
	BL6	BL6	TLR2^−/−^
Body end weight (g)	31.8 ± 0.5	35.7 ± 1.2	36.5 ± 1.4
Liver/body weight ratio (%)	5.49 ± 0.1	5.14 ± 0.2	5.27 ± 0.2
Triglycerides (mg)/ protein (mg)	3.87 ± 0.3	14 ± 3.1	12 ± 2.1
AST (U/L)	19.7 ± 5.4	46.3 ± 4.0	42.3 ± 2.7
*p21* mRNA expression (% of 4mo BL6 mice)	100 ± 12	222 ± 32	210 ± 40
Xylose permeation (mmol/L)	0.25 ± 0.03	0.95 ± 0.13	1.12 ± 0.11
TLR2 ligands (OD655nm)	0.17 ± 0.01	0.20 ± 0.01	0.25 ± 0.03

Data presented as mean ± SEM, *n* = 7–10

*AST* aspartate aminotransferase, *BL6 *C57BL/6J, *TLR2*^*−/−*^ TLR2 Knockout

*p*-value determined by unpaired *t*-test (body weight, triglycerides and AST, p21 mRNA expression, xylose permeation) and Mann–Whitney *U* test (Liver/body weight ratio, TLR2 ligands)

### Western blot

Plasma from the retrobulbar plexus was loaded on polyacrylamide gels at a concentration of 0.8 µg/µl. After electrophoresis, protein was transferred to a polyvinylidene difluoride membrane (Bio-Rad Laboratories, USA). Membranes were blocked and incubated with the primary antibody C-reactive protein (CRP; Santa Cruz, USA), overnight followed by an incubation with the respective secondary antibody for 1.5 h at room temperature. Bands were detected using Super Signal West Dura kit (Thermo Fisher Scientific, USA) as detailed previously [14]. Densitometric analysis of bands was performed using the Software Image Lab 6.1 (Bio-Rad Laboratories, USA), and protein bands were normalized to ponceau.

### UMAP visualization

The UCSC Cell Browser (https://cells.ucsc.edu/) analyzing liver cell dataset of the single cell transcriptomic atlas “Tabula muris senis” characterizing aging tissue in mouse was used to create Uniform Manifold Approximation and Projection (UMAP) visualization [35, 36]. They performed single cell RNA sequencing in male and female C57BL/6 mice with an age ranging from 1 month to 30 months of age [35].

### Statistical analysis

PRISM (Version 7.03, GraphPad Software, Inc.) was used for statistical analysis. Outliers were determined with Grubb´s test. After testing for Gaussian distribution, unpaired *t*-test or Mann–Whitney *U* test was used to determine statistical differences between two groups. Bartlett’s test was applied to test homogeneity of variances and data were log-transformed if unequal before performing one-way or two-factorial analysis of variance (ANOVA) followed by Tukey´s Post Hoc test to compare four groups. The number of biological replicates and independent experimental repeats is indicated in each figure legend corresponding to each experiment. All data are presented as means ± standard error of means (SEM). *p* < 0.05 was defined to be significant.

## Results

### TLR2/2, TLR2/1, and TLR2/6 ligands in healthy young and elderly humans and mice

**Fig. 1:**
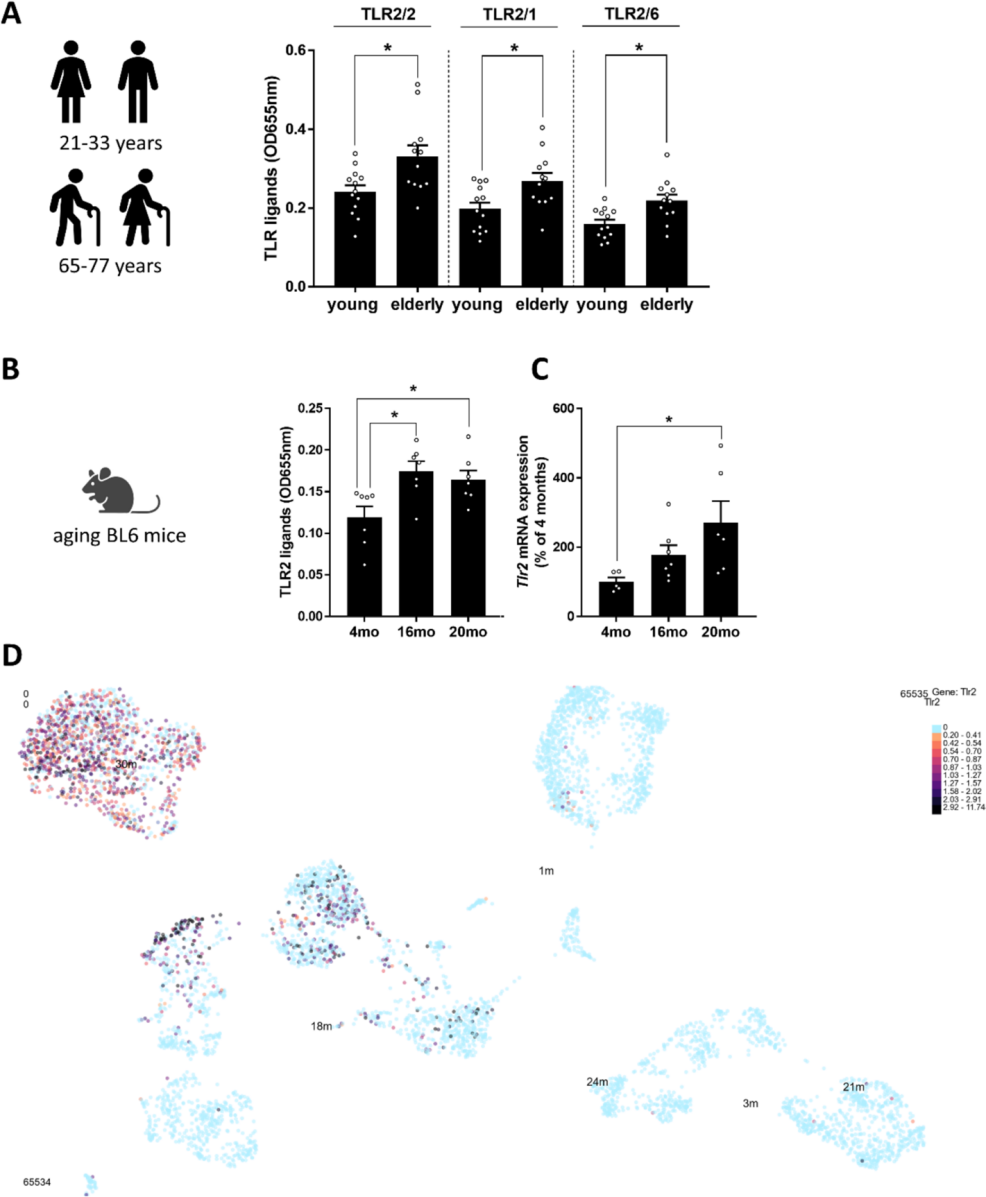
TLR2 ligands concentration in aging humans and mice as well as *Tlr2* mRNA expression in liver tissue. **A** Figure of study cohort and Toll-like receptor (TLR) 2/2, 2/1, and 2/6 ligands in serum of young and elderly healthy subjects. **B** Figure of model organism and TLR2 ligands in plasma as well as **C** *Tlr2* mRNA expression in liver of 4 months (mo), 16 mo, and 20 mo old male C57BL/6J (BL6) mice. **D** UMAP visualization of single-cell RNA-seq data showing *Tlr2* gene expression across different ages in liver on the basis of the Tabula Muris Senis liver single-cell RNAseq 10 × Droplet Dataset. Each point represents an individual data point, colored according to the expression level of *Tlr2*. Clusters labeled with age indicate samples collected at different ages. High expression of *Tlr2* is shown in dark colors, while low expression is in light blue. Data are presented as mean ± SEM, A: *n* = 12–13, B-C: *n* = 5–7, *p < 0.05. *p*-values were determined by A: unpaired *t*-test, B-C: one-way analysis of variance (ANOVA) followed by Tukey´s Post Hoc test

Although elderly participants (mean age 71.4 years) did not present with overt diseases, including liver disorders—as indicated by alanine aminotransferase (ALT) and AST levels comparable to those of young, healthy controls—concentrations of TLR2/2, TLR2/1, and TLR2/6 ligands were significantly elevated in the elderly group (Fig. 1A, Table 1). Similarly, aged mice (16 and 20 months old) exhibited significantly higher levels of TLR2 ligands in the plasma compared with young animals (Fig. 1B). Furthermore, hepatic *Tlr2* mRNA expression appeared to increase progressively with age, reaching significantly higher levels in 20-month-old mice than in young controls (Fig. 1C). Consistent with these findings, UMAP visualization of single-cell RNA-seq data from the Tabula Muris Senis liver dataset, accessed via the UCSC Cell Browser, revealed increased *Tlr2* expression across various liver-associated cell types with age (Fig. 1D) [35, 36].

### Effect of a genetic deletion of TLR2 in mice on markers of age-associated senescence, liver damage, and intestinal barrier function

**Fig. 2:**
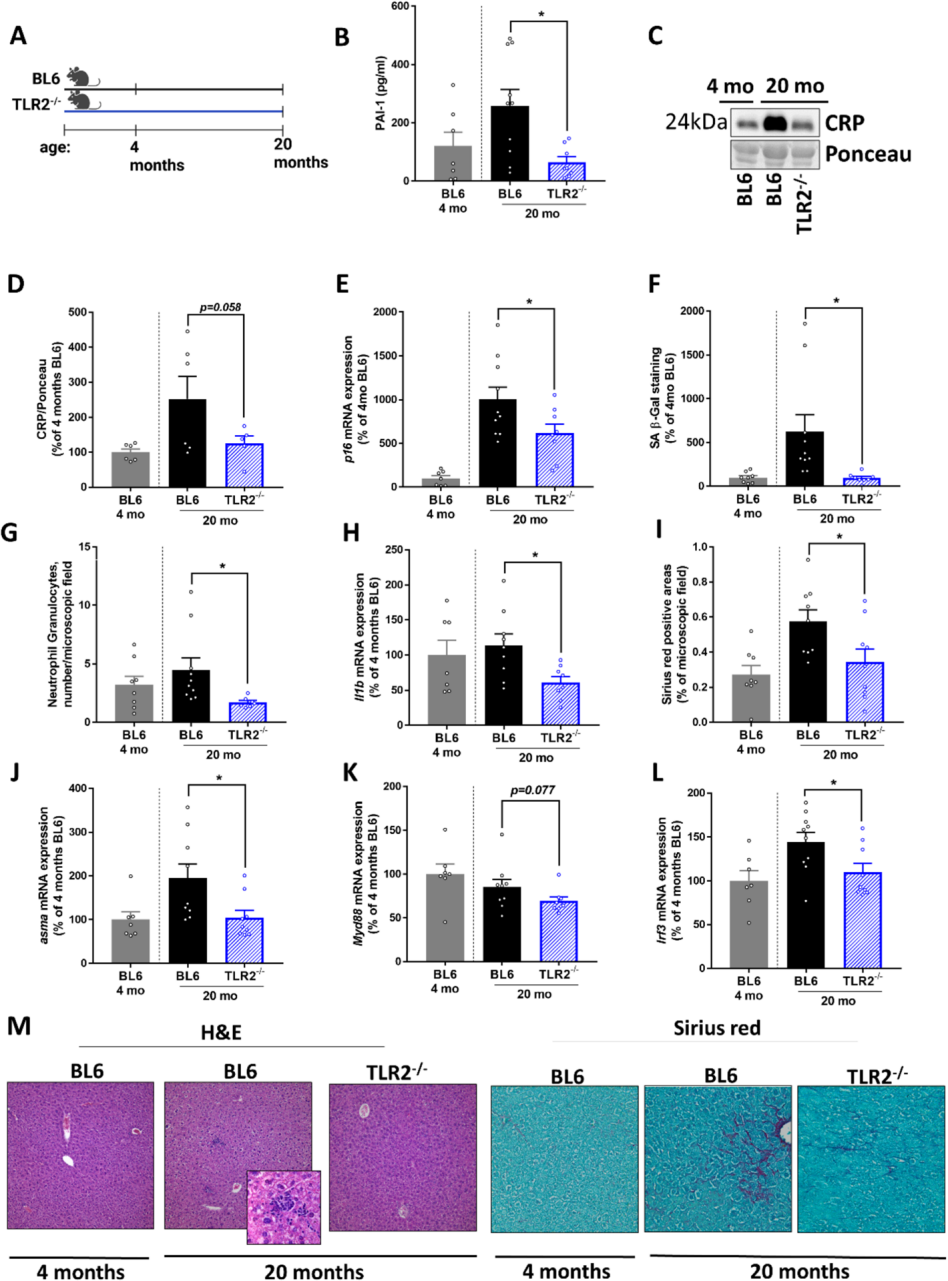
Markers of senescence and liver damage in plasma and liver of 4-month and 20-month-old BL6 and TLR2^−/−^ mice. **A** Study design, **B** plasminogen activator inhibitor-1 (PAI-1) concentration in plasma and **C** representative western blots as well as **D** densitometric analysis of C-reactive protein (CRP) in plasma. **E ***p16* mRNA expression in liver, **F** senescence-associated (SA) β-galactosidase staining in liver sections, **G** number of neutrophil granulocytes in liver sections, **H ***interleukin 1 beta* (*Il1b*) mRNA expression in liver, **I** Sirius red positively stained areas in liver sections and **J ***alpha smooth muscle actin* (*asma*) as well as **K ***myeloid differentiation primary response 88* (*MyD88*) and **L ***interferon regulatory factor 3* (*Irf3*) mRNA expression of hepatic tissue. **M** Representative pictures of hematoxylin and eosin-stained liver Sections (100×, 630×) and Sirius red stained Sects. (200×) in 4-month and 20-month-old BL6 and TLR2^−/−^ mice. Data are presented as mean ± SEM, *n* = 7–10, except for western blot analysis D: *n* = 6. *p < 0.05. BL6–C57BL/6J, TLR2^−/−^-TLR2 Knockout. *p*-values were determined by B, D–E, H–L: unpaired *t*-test, F + G: Mann–Whitney *U* test

To assess the effects of a genetic deletion of TLR2 on markers of senescence and liver damage during aging, 20-month-old TLR2^−/−^ mice were compared with age-matched wild-type mice (Fig. 2A). PAI-1, and CRP protein levels, markers that have been shown to be indicative of senescence and inflammaging [37, 38], were significantly and by trend (CRP,* p* = 0.058) higher in plasma of 20-month-old C57BL/6J mice when compared with age-matched TLR2^−/−^ mice (see Fig. 2B–D). Indeed, while only shown for comparison, concentrations of PAI-1 and CRP protein in plasma of old aged TLR2^−/−^ mice was almost at the level of young naive wild-types. Expression of *p16* mRNA and SA-associated β-gal staining were also significantly lower in livers of 20-month-old TLR2^−/−^ mice when compared with 20-month-old C57BL/6J mice (Fig. 2E–F, Supplementary Fig. 2), while *p21* mRNA expression in liver was similar between aged mouse groups (Table 2). Neither body weight, nor liver/body weight ratio or hepatic triglyceride concentration and AST activity in plasma differed between 20-month-old C57BL/6J and TLR2^−/−^ mice (Table 2). Livers of 20-month-old C57BL/6J mice displayed notable signs of inflammation and beginning fibrosis, which were markedly less prominent in livers of TLR2^−/−^ mice (Fig. 2M). Specifically, numbers of neutrophil granulocytes and *interleukin 1b* (*Il1b*) mRNA expression in livers were significantly higher in 20-month-old C57BL/6J mice than in age-matched TLR2^−/−^ mice (Fig. 2G–H). Also, signs of hepatic fibrosis, as determined by Sirius red staining and *alpha smooth muscle actin* (*asma*) mRNA expression in liver were both significantly lower in TLR2^−/−^ mice compared with C57BL/6J mice (Fig. 2I-J). The protective effects found for the genetic deletion of TLR2 in aging mice were related with lower mRNA expression of *myeloid differentiation primary response 88* (*Myd88*) and *interferon regulatory factor 3* (*Irf3*) in hepatic tissue (*Myd88*: *p* = 0.077, *Irf3*: *p* < 0.05) when compared with age-matched wild-type mice (Fig. 2K–L).

To assess the impact of a genetic deletion of TLR2 on age-related impairments of intestinal barrier function permeation of xylose was assessed ex vivo on everted small intestinal tissue sac and TLR2 ligands were measured in plasma. There were no differences in either xylose permeation in small intestinal tissue or TLR2 ligand concentration in plasma between 20-month-old TLR2^−/−^ mice and age-matched wild-type mice (Table 2).

### Effect of LTA on markers of senescence in J774A.1 cells and human PBMCs from healthy young donors

**Fig. 3:**
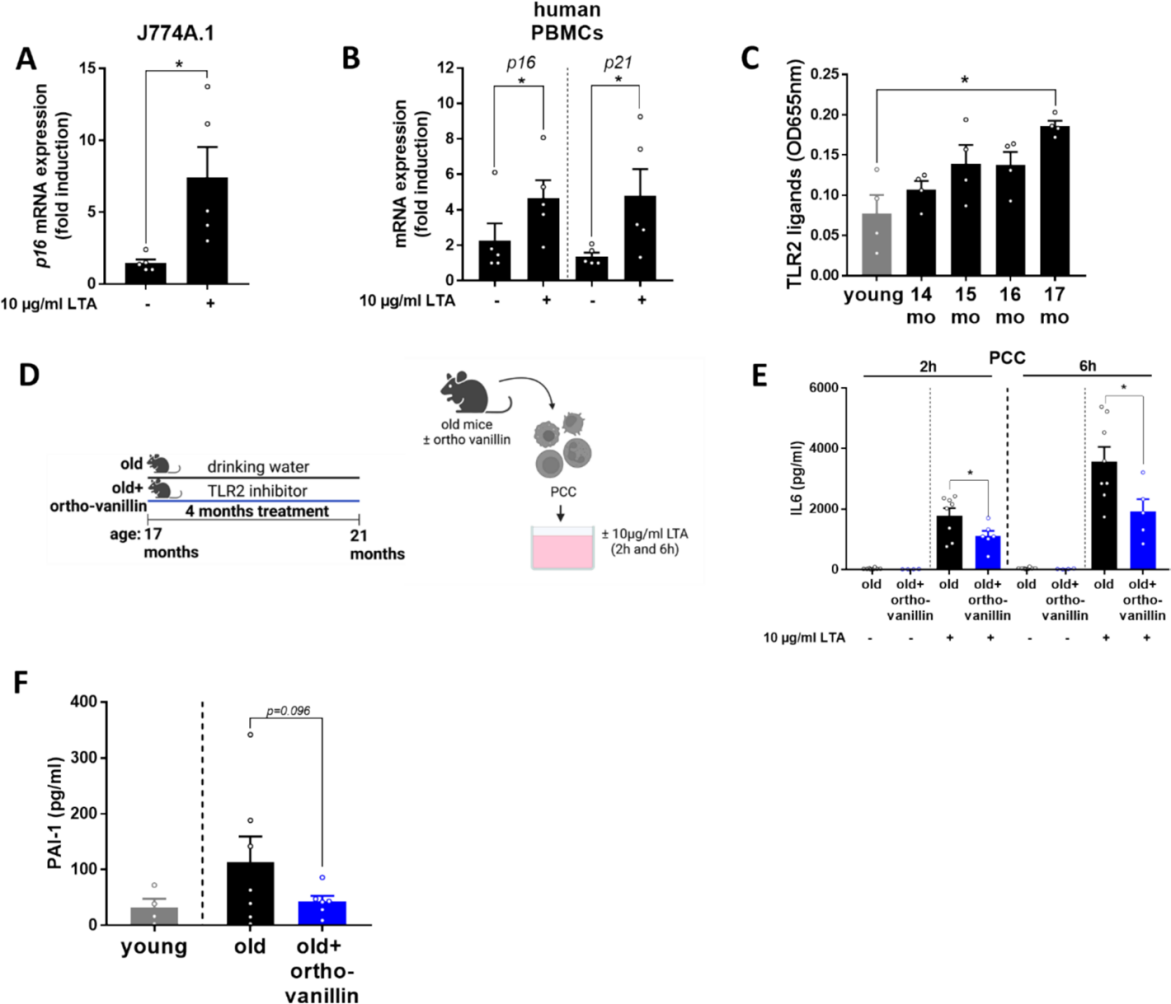
Effects of LTA and the TLR2 inhibitor ortho-vanillin on markers of senescence in immune cells and mice. **A ***p16* mRNA expression in J774A.1 cells incubated with 10 µg/ml LTA for 6 h, **B ***p16* and *p21* mRNA expression in human PBMCs stimulated with 10 µg/ml LTA for 48 h. **C** Toll-like receptor 2 (TLR2) ligand levels in peripheral blood of aging mice. **D** Study design of mouse experiment and experimental protocol of peritoneal cavity cells (PCCs) incubation. **E **Interleukin 6 (IL6) protein concentration in cell culture supernatant of LTA-stimulated (10 µg/ml) PCCs isolated from mice treated with ± ortho-vanillin. **F** Plasminogen activator inhibitor-1 (PAI-1) protein concentration in plasma of aging mice treated with ± 60 mg/kg BW ortho-vanillin via drinking water. Data are presented as mean ± SEM, A–B: *n* = 5, C: *n* = 4 (pooled samples), **E ***n* = 4–8, **F** *n* = 6–8, except PAI-1 in young mice: *n* = 4 due to levels lower than the detection limit, *p < 0.05. *p*-values were determined by A: unpaired *t*-test, **B** (p16): Mann–Whitney *U* test, **B** (p21): unpaired *t*-test, **C** one-way ANOVA followed by Tukey´s Post Hoc test, **E**–**F** unpaired t-test

The UMAP visualization of the single cell RNA seq data (UCSC Cell Browser [35, 36]) revealed *Tlr2* gene expression to be particularly enriched in immune-related clusters such as Kupffer cells and myeloid leukocytes (Supplementary Fig. 1). To determine whether TLR2 ligands are direct regulators of senescence in immune-related liver cells, murine J774A.1 cells, which are frequently used as a model of hepatic Kupffer cells, were challenged with LTA for 6 h. Expression of *p16* mRNA in cells challenged with LTA was significantly higher than in naive cells (Fig. 3A). To further determine whether LTA affects expression of senescence markers in humans as well, PBMCs, being a model of immune cells, isolated from healthy young adults were challenged with 10 µg/ml LTA for 48 h. In line with the findings in murine J774A.1 cells, *p16* as well as *p21* mRNA expression were also increased in LTA-stimulated human PBMCs (Fig. 3B).

### Effect of inhibiting TLR2 with ortho-vanillin in aging mice on markers of senescence and liver damage

To further determine if an inhibition of TLR2 may also attenuate the progression of age-related liver decline in old age, 17-month-old, male C57BL/6J mice showing significantly increased concentrations of TLR2 ligands in peripheral plasma (Fig. 3C), compared with young mice, were treated with the TLR2 inhibitor ortho-vanillin for 4 months (Fig. 3D). Ortho-vanillin is a small molecule that has been reported to inhibit TLR2 signaling by targeting a BB loop within the TLR2 TIR domain [39], without altering TLR4 signaling [40] and is reported to have senolytic and senomorphic properties [41, 42]. Using PCCs isolated from mice treated with the TLR2 inhibitor, we found that the LTA-stimulated (10 µg/mL, 2 and 6 h stimulation) release of IL6 was significantly lower by ~38% and ~46% in cells isolated from mice ingesting drinking water enriched with the TLR2 inhibitor compared with those obtained from mice that were not treated with the inhibitor (Fig. 3E).

**Fig. 4:**
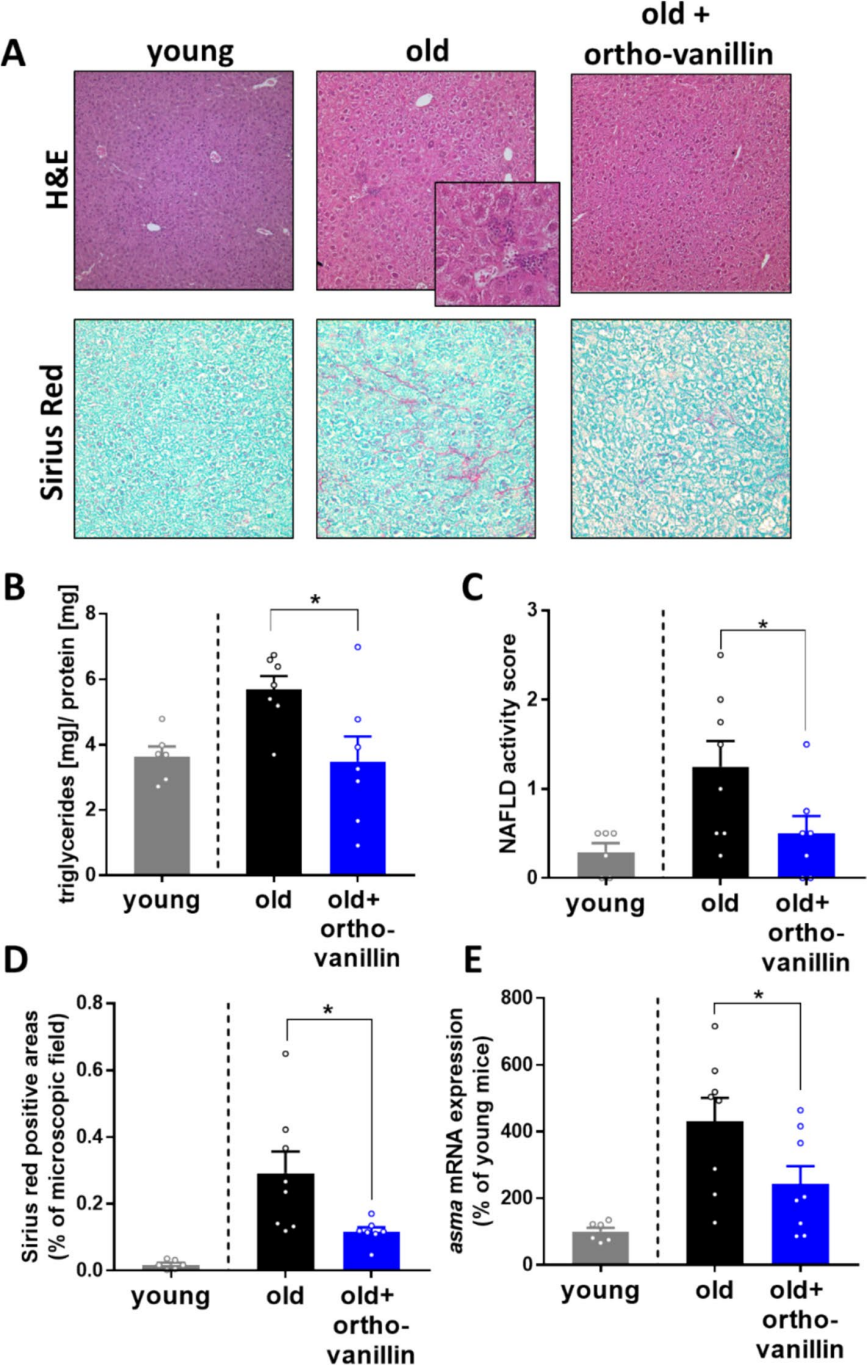
Effect of the treatment with TLR2 inhibitor ortho-vanillin on markers of liver damage in aging mice. **A** Representative pictures of hematoxylin and eosin (H&E, 100×, 400×) and Sirius red stained liver sections (200×). **B** Triglyceride concentration in liver, **C** NAFLD activity score, **D** Sirius red positive-stained areas in liver sections and **E** *alpha smooth muscle actin* (*asma*) mRNA expression of hepatic tissue in aging mice treated with ± 60 mg/kg BW ortho-vanillin via drinking water. Data are presented as mean ± SEM, *n* = 6–8, *p < 0.05. *p*-values were determined by B–E: unpaired *t*-test

In the plasma of mice treated with the TLR2 inhibitor, PAI-1 protein concentrations showed a trend (*p* = 0.096) towards being lower compared with aged-matched vehicle controls (Fig. 3F). Neither body weight nor liver/body weight ratio or AST activity in plasma differed between groups. However, the triglyceride concentration in liver tissue was significantly lower in the TLR2 inhibitor-treated mice compared with age-matched vehicle controls (Fig. 4B, Table 3). The number of inflammatory foci in the liver tissue of old aged mice treated with the TLR2 inhibitor was significantly lower than that in vehicle controls, while the number of neutrophil granulocytes in liver sections was similar between groups (Fig. 4A,C, Table 3). Furthermore, signs of hepatic fibrosis were also markedly less pronounced in mice treated with the inhibitor. Specifically, Sirius red-stained positive areas and *asma* mRNA expression were both significantly lower in livers of aging mice treated with the TLR2 inhibitor ortho-vanillin compared with mice receiving vehicle (Fig. 4A,D–E). While *p16* and *p21* mRNA expression did not differ in the livers of age-matched mice treated with or without ortho-vanillin, SA β-gal staining being also a marker of senescence, was by trend lower in ortho-vanillin-treated mice compared with controls (*p* = 0.053, Table 3, Supplementary Fig. 2).

**Table 3:** Effect of the treatment with the TLR2 inhibitor ortho-vanillin on body weight and markers of liver damage in aging mice

Parameter	Groups		
	4 months	21 months	
	young	old	old + ortho-vanillin
Body end weight (g)	28.2 ± 1.2	35.1 ± 0.6	34.7 ± 0.5
Liver/body weight ratio (%)	5.8 ± 0.2	5.1 ± 0.1	5.4 ± 0.2
Neutrophil granulocytes (number/microscopic field)	0.4 ± 0.1	2.7 ± 0.4	3.0 ± 0.5
AST (U/L)	28.6 ± 4.9	42.0 ± 3.5	35.3 ± 3.5
SA β-gal staining (% of young mice)	100 ± 28	921 ± 202	324 ± 149
*p16* mRNA expression (% of young mice)	100 ± 18.6	1171 ± 245	2099 ± 962
*p21* mRNA expression (% of young mice)	100 ± 12	372 ± 79	404 ± 99

Data are presented as mean ± SEM, *n* = 6–8

*AST* aspartate aminotransferase, senescence-associated (SA) β-galactosidase staining assessed in liver sections

*p*-value determined by unpaired t-test and Mann–Whitney *U* test (SA β-gal, p16, p21)

### Effect of a genetic deletion of TLR2 on development of diet-induced MASLD

To further determine if TLR2 is critical in hepatic fat accumulation, we pair-fed young, female C57BL/6 as well as TLR2^−/−^ mice with a fat-, fructose-, and cholesterol-rich diet (FFC-diet) for 6 weeks to induce MASLD. While body weight and liver/body weight ratio as well as fat accumulation assessed by liver histology and *perilipin-2* were similar between FFC-fed groups, NAFLD activity score was significantly lower in FFC-fed TLR2^−/−^ mice compared with FFC-fed BL6 mice (Supplementary Table 3, Supplementary Fig. 3A–C). In line with these findings, hepatic *Il1b* and *F4/80* mRNA expression was also significantly lower in FFC-fed TLR2^−/−^ mice compared with FFC-fed BL6 mice, suggesting that the loss of TLR2 predominantly impacted hepatic inflammation (Supplementary Fig. 3D–E).

## Discussion

Studies suggest that old age is an independent risk factor for the development of various metabolic diseases, including liver diseases such as MASLD [43]. Here, employing studies in healthy elderly humans and mice, we demonstrate that healthy aging in humans and mice is associated with higher concentrations of TLR2 ligands in blood and higher *Tlr2* mRNA expression in liver tissue. Moreover, the concentration of TLR2 ligands in portal plasma seemed to remain constant after an initial increase around the age of 16 months. In contrast, mRNA expression of *Tlr2* in liver tissue seems, after an initial increase paralleling the increase of TLR2 ligand concentration in portal blood to further increase with age. It has been shown before in various species, including humans, that aging is associated with an altered intestinal microbiota composition. Indeed, the intestinal microbiota dysbiosis is now recognized as a hallmark of aging [44] being closely related with impaired intestinal barrier function (for an overview see [45]). The latter alteration has been linked to an increased translocation not only of bacterial endotoxins derived from Gram-negative bacteria but also bacterial toxins originating from the outer wall of Gram-positive bacteria [14]. In line with the increase of bacterial toxins accounted to Gram-positive bacteria in portal blood, an upregulation of *Tlr1*, *2* and *6* mRNA has been reported in various tissues, including the liver [9, 14]. In the present study, HEK cells stably transfected with TLR2 were employed to assess TLR2 ligands in mouse and human plasma. TLR2 has been shown to also form heterodimers with TLR1 and TLR6 and to recognize a rather heterogeneous group of bacterial (*e.g*., peptidoglycan, LTA) and even viral compounds (hemagglutinin from measles virus), which may differentially impact *Tlr2* mRNA expression and TLR2-dependent signaling cascades [19, 46]. It is also possible that age-related changes in the intestinal microbiota composition alter the composition of TLR2 ligands, which in turn may affect *Tlr2* expression. This needs to be clarified in further studies. Taken together, our results provide additional support for the hypothesis that even healthy aging, both in humans and rodents, is related to an increased permeation of Gram-positive bacteria-derived toxins and an induction of *Tlr2* expression in liver tissue.

### Blocking TLR2 alters markers of senescence and inflammation

In settings of healthy aging, markers of senescence and inflammation such as p16 and PAI-1 along with CRP are elevated [37, 38, 47]. In the present study, both the genetic deletion of TLR2 and the treatment of animals with the TLR2 inhibitor ortho-vanillin were associated with lower levels of markers and mediators of senescence and inflammation in blood. Analysis of scRNAseq data obtained from aging mice, analyzing the liver cell dataset of the single cell transcriptomic atlas “Tabula muris senis” [35, 36] revealed that TLR2 is predominantly expressed in immune cells in liver tissue. Moreover, in J774A.1 cells and human PBMCs challenged with the TLR2 ligand LTA, mRNA expression of the senescence markers *p16* and *p21* were significantly induced. Studies of others suggest that senescence in fibroblasts may be linked to high expression of TLR2 [20]. Supporting the hypothesis that TLR2 and its ligand LTA may be critical in the regulation of senescence, it has also been shown, that challenging bone marrow-derived macrophages with low doses of LTA (1 µg/ml) may abolish signs of senescence related to bone degeneration in mice [48], further suggesting that effects of LTA may be related to dosing. In summary, our data suggest that blocking TLR2 in healthy aging mice might reduce mediators of senescence and inflammation and that high doses of LTA can induce markers of senescence in immune cells. These results by no means preclude that other factors are also critical in the development of senescence and inflammaging, but rather suggest that an activation of TLR2 may at least contribute at some stages to the aging process. Also, it remains to be determined which of the TLR2 ligands is critical herein or if at least some of the effects are also a question of dosage.

### Blocking TLR2 dampened age-associated liver damage in mice

Aging is associated with systemic chronic inflammation (also referred to as “inflammaging”) and cellular senescence, contributing to liver decline even in the absence of disease-dependent triggers (e.g., overweight, insulin resistance, viruses) [18, 49]. Indeed, it has been shown that senescent cells accumulate across various hepatic cell types and shape hepatic dysfunction through the proinflammatory components of their senescence-associated secretory phenotype (SASP) [50, 51]. In this study, both genetic deletion of TLR2 and pharmacological inhibition using ortho-vanillin in wild-type mice exhibiting early signs of age-related organ dysfunction (e.g., impaired intestinal barrier function) were associated with a marked reduction in age-related inflammatory and fibrotic changes in liver tissue. While a genetic deletion of TLR2 has been shown before to be related to a protection of animals from age-associated exacerbation of the cardiac inflammatory response [52], to the best of our knowledge, this is the first study assessing the role of TLR2 in age-related liver senescence. Indeed, blocking TLR2 protected mice from age-associated induction of hepatic senescence markers. However, while *p16* mRNA and SA-associated β-gal staining were clearly downregulated in aged TLR2^−/−^ mice, *p21* mRNA expression was not altered. Senescent cells commonly upregulate the cell cycle inhibitors p21 and p16, thereby driving cell cycle arrest. However, respective senescent cells might represent distinct cell populations with different types, locations, accumulation kinetics, and functions [53]. Comprehensive analysis of different cell types and tissues indicate that *p21*^*Cip1*^, and *p16*^*Ink4a*^ mRNA expression varies markedly among cell types and tissues and are often not coexpressed [54]. Further studies are needed to determine the expression pattern of *p21*^*Cip1*^, and *p16*^*Ink4a*^ in different liver cells of aging C57BL/6 mice. Consistent with previous findings in cardiac tissue, aged TLR2^−/−^ mice and aged wild-type mice treated orally with ortho-vanillin exhibited significantly reduced inflammatory changes as well as attenuated signs of hepatic fibrosis. The latter was shown in the present study but also in studies of others to attenuate the LTA-dependent activation of TLR2-dependent signaling cascades in immune cells [39]. Moreover, while *Myd88* expression was slightly decreased (*p* = 0.077), expression of *Irf3* mRNA was lower in livers of TLR2^−/−^ mice. It has been suggested that an activation of IRF3 may be critical in senescence and may mitigate liver fibrosis [55]. It has also been discussed that the TLR2-dependent activation of IRF3 is only found in subsets of inflammatory monocytes (CD11c^−^CD11b^+^Ly6C^+^) [56, 57]. Indeed, others have shown that CD11b + Ly6C + myeloid cells are crucial for the progression of metabolicdysfunction-associated steatohepatitis [58]. Further studies are needed to determine how TLR2 may “selectively” activate IRF3 and the role of IRF3 in age-related (liver) degeneration. Interestingly, while the triglyceride levels in the livers of aged TLR2^−/−^ and wild-type mice were similar, the treatment of aging wild-type mice with the TLR2 inhibitor was related to a lower triglyceride concentration in liver. Somewhat in line with these findings, inflammatory alterations were markedly attenuated in TLR2^−/−^ mice fed a fat-, fructose-, and cholesterol-rich diet while fat accumulation as detected by liver histology and assessment of *perilipin-2* was similar between groups. It could be that ortho-vanillin, while being described as a specific inhibitor of TLR2 [40] may also have other so far unknown effects. This needs to be determined in future studies. In summary, our findings indicate that TLR2 inhibition in healthy aged mice may reduce the propagation of senescent cell burden by dampening the SASP.

### Blocking TLR2 did not affect age-associated impairments of intestinal barrier function

Several studies suggest that even in settings of healthy aging, age-related liver decline is associated with impairments of intestinal barrier function and an altered microbiota composition [9, 12, 59]. Indeed, in results of previous studies both markers of intestinal permeability and bacterial toxins concentration in blood were higher in old aged humans and mice, even in the absence of any disease driving factors [9, 12, 14, 60]. However, it is still unclear if and how an activation of TLR2 directly influences the intestinal barrier function. Studies in models of IBD suggest that an impairment of TLR2 signaling might add to an imbalance of the commensal-dependent intestinal epithelial barrier defense [61]. Other studies suggest that mice lacking TLR2 exhibited early tight junction disruption in experimental models of colitis [62]. Results of the present study suggest that a genetic deletion of TLR2 does not affect age-related impairments to intestinal barrier function as concentrations of TLR2 ligands were similar between age-matched mouse groups. Rather, results of our studies suggest that the protective effects on age-related liver damage and senescence of a genetic deletion or chemical inhibition of TLR2 may stem from the inhibition of the TLR2-associated proinflammatory signaling cascade in liver tissue.

## Conclusions

Our data further support the hypothesis that even in healthy aging, the induction of TLR2-dependent signalling cascade plays a critical role in the development and progression of age-related organ decline, particularly in the liver. Moreover, our data also suggest that targeting TLR2 signalling may slow the process in mice. Indeed, recent studies suggest that ortho-vanillin, the TLR2 inhibitor used in the present study, may possess senolytic and senomorphic properties [41, 42]. However, further research is needed to assess whether TLR2 and its associated signaling cascades are also critical in human aging, and whether this is affected by diseases (*e.g*., metabolic diseases) which are associated with higher bacterial toxin levels. In addition, further studies in both humans and mice are needed to assess whether targeting TLR2 might represent a therapeutic strategy for age-associated liver decline.

## Supplementary Information

Supplementary Material 1.

Supplementary Material 2.

## Data Availability

The original contributions presented in the study are included in the article/ Supplementary Material, further inquiries can be directed to the corresponding author.

## Abbreviations

Αsma: Alpha smooth muscle actin
ANOVA: Analysis of variance
AST: Aspartate aminotransferase
C-diet: Control diet
CD14: Cluster of differentiation 14
CRP: C-reactive protein
FBS: Fetal bovine serum
FFC-diet: Fat-, fructose- and cholesterol-rich diet
IL: Interleukin
IRF: Interferon regulatory factor
LBP: Lipopolysaccharide binding protein
LTA: Lipoteichoic acid
MASLD: Metabolic dysfunction-associated steatotic liver disease
Mo: Months
MyD88: Myeloid differentiation primary response 88
NFκB: Nuclear factor kappa-light-chain-enhancer of activated B cells
PAI-1: Plasminogen activator inhibitor-1
PAMP: Pathogen-associated molecular patterns
PBMC: Peripheral blood mononuclear cells
PCC: Peritoneal cavity cells
PCR: Polymerase chain reaction
PRR: Pattern recognition receptor
SEM: Standard error of means
TIRAP: Toll-interleukin-1 receptor domain-containing adaptor protein
TLR: Toll-like receptor
