# Supplementary material for: Toll-like receptor 2 drives liver senescence and fibrosis in aging through gut-derived microbial signaling
Source: Cell Mol Biol Lett. 2026 Mar 3;31:50. doi: 10.1186/s11658-026-00881-4 (PMC13064244; doi:10.1186/s11658-026-00881-4)
Supplement: Supplementary file 1 — Supplementary Material 1. [file 11658_2026_881_MOESM1_ESM.pdf]

Full unedited blots

Toll-like receptor 2 drives liver senescence and fibrosis in aging through gut-derived microbial signaling

Annette Brandt[1], Raphaela Staltner[1], Anja Baumann[1], Katharina Burger[1], Julia Jelleschitz[2], Patricia De Oliveira Prada[1], Annika Höhn[2,3], Florian Kopp[4], Jordi Mayneris-Perxachs[5], José Manuel Fernández-Real[6], Ina Bergheim[1#]

Corresponding author:
Ina Bergheim, Ph.D.
University of Vienna
Department of Nutritional Sciences
Molecular Nutritional Science
Josef-Holaubek-Platz 2 (UZA II)
A-1090 Wien
Phone: +43-1-4277-549 81
E-Mail: ina.bergheim@univie.ac.at

Full unedited Blots Figure 2C – CRP

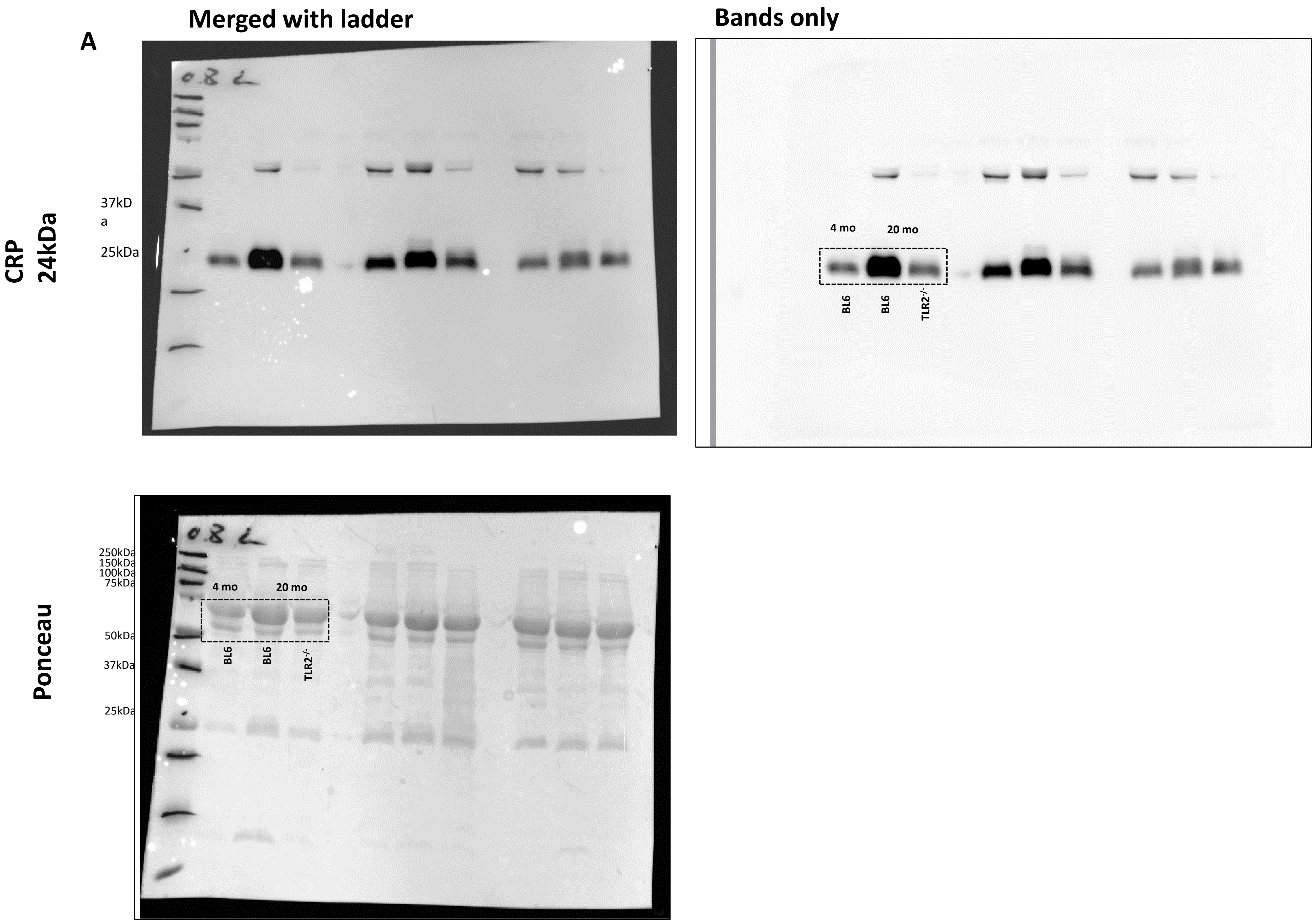

Source Data 1: Original blots of Figure 2C (CRP). Original blots of C-reactive protein (CRP) (Left side: Bands merged with ladder, Right side: Bands only) and ponceau-stained blot. Bands that are shown in Figure 2C are highlighted with a frame.
