# Supplementary material for: Toll-like receptor 2 drives liver senescence and fibrosis in aging through gut-derived microbial signaling
Source: Cell Mol Biol Lett. 2026 Mar 3;31:50. doi: 10.1186/s11658-026-00881-4 (PMC13064244; doi:10.1186/s11658-026-00881-4)
Supplement: Supplementary file 2 — Supplementary Material 2. [file 11658_2026_881_MOESM2_ESM.pdf]

Supplemental Figures and Tables

Toll-like receptor 2 drives liver senescence and fibrosis in aging through gut-derived microbial signaling

Annette Brandt[1], Raphaela Staltner[1], Anja Baumann[1], Katharina Burger[1], Julia Jelleschitz[2], Patricia De Oliveira Prada[1], Annika Höhn[2,3], Florian Kopp[4], Jordi Mayneris-Perxachs[5], José Manuel Fernández-Real[6], Ina Bergheim[1#]

Corresponding author:
Ina Bergheim, Ph.D.
University of Vienna
Department of Nutritional Sciences
Molecular Nutritional Science
Josef-Holaubek-Platz 2 (UZA II)
A-1090 Wien
Phone: +43-1-4277-549 81
E-Mail: ina.bergheim@univie.ac.at

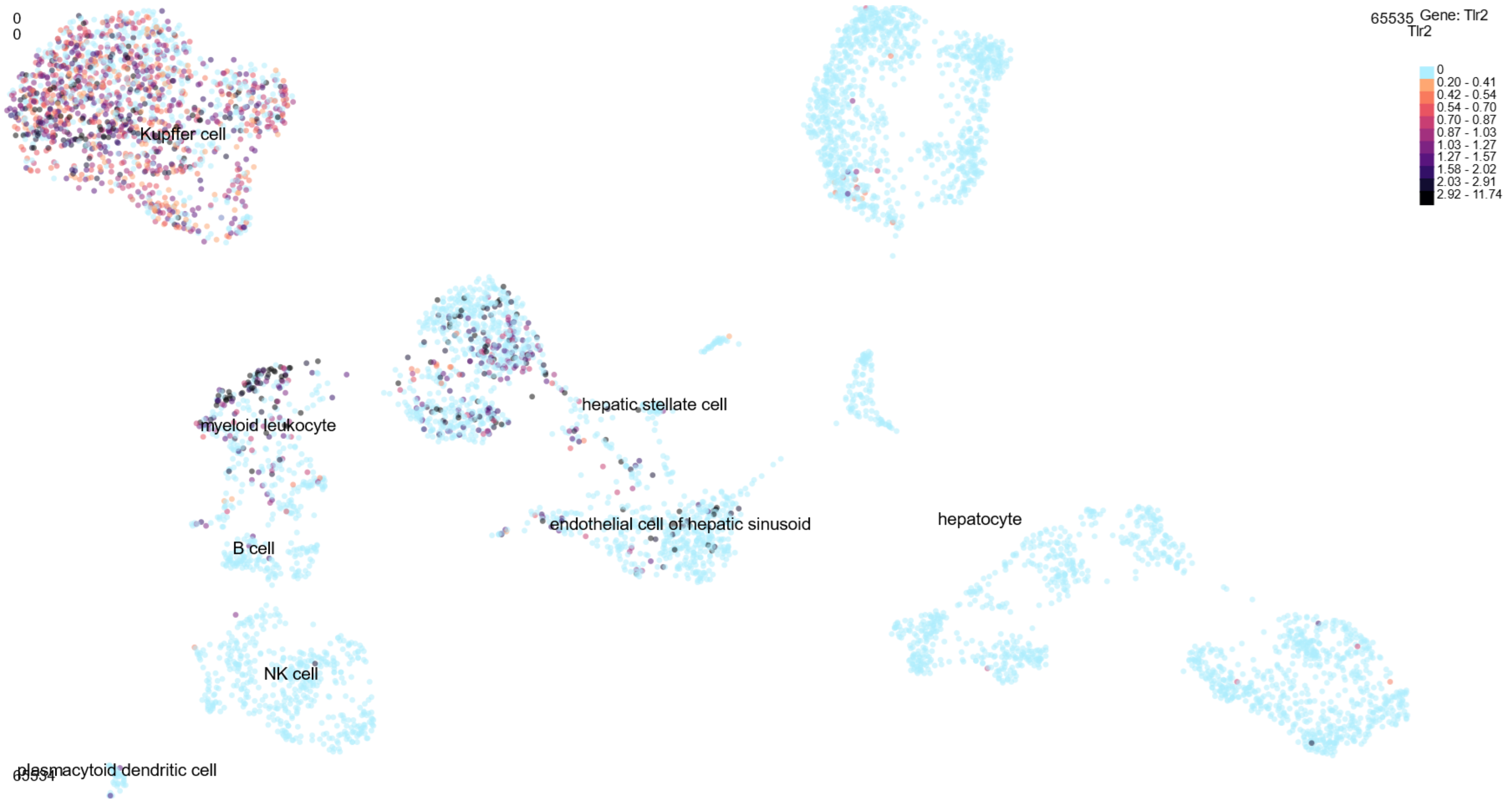

Supplemental Figure 1: UMAP visualization of single-cell RNA-seq data showing *Tlr2* gene expression across liver-associated cell types. UMAP visualization of single-cell RNA-seq data showing *Tlr2* expression across liver-associated cell types. Each point represents an individual cell, and colors indicate *Tlr2* expression levels. Major cell populations, including Kupffer cells, hepatocytes, endothelial cells, B cells, NK cells, and myeloid leukocytes, are labeled. Darker colors denote higher *Tlr2* expression.

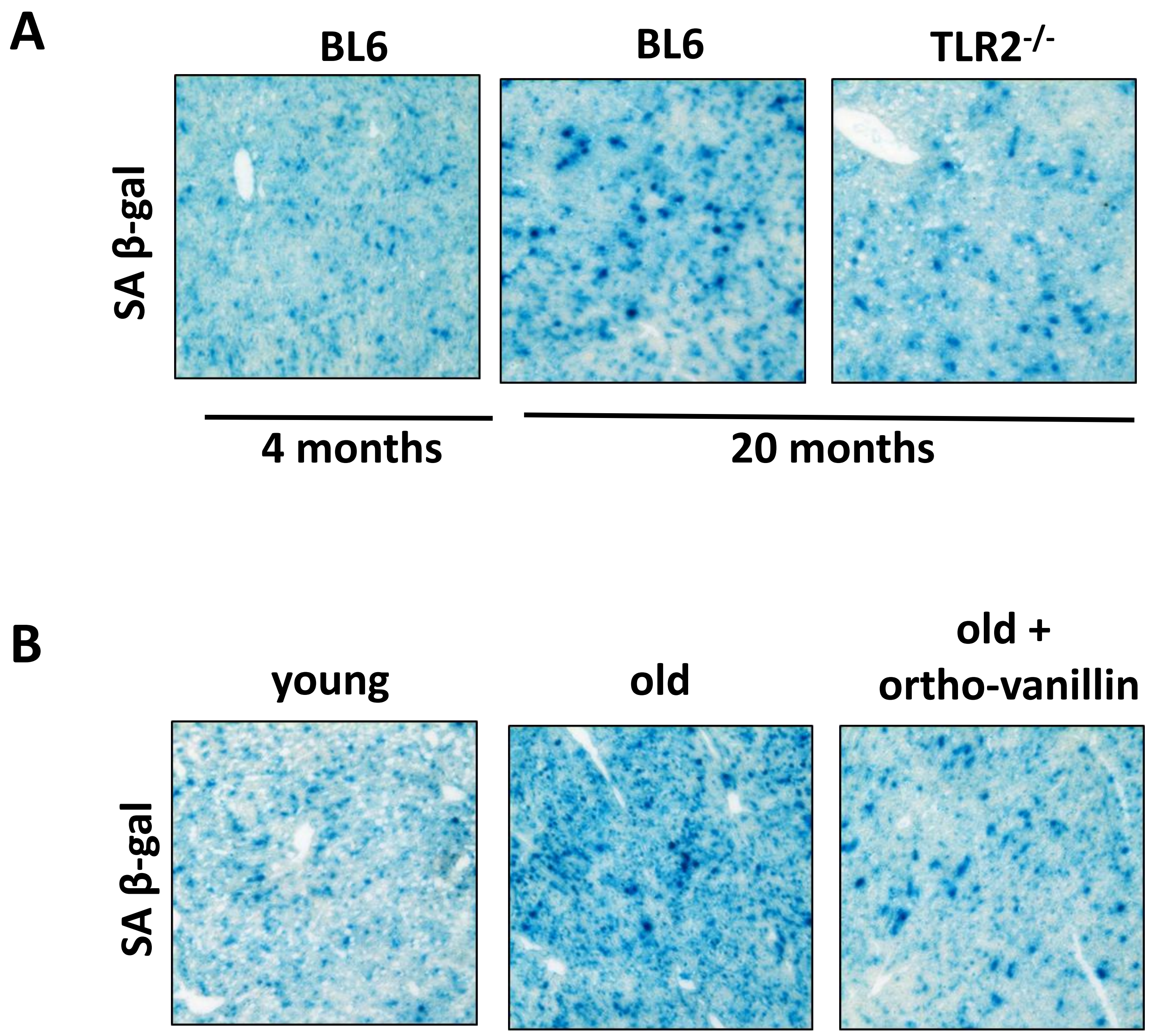

Supplemental Figure 2: Representative pictures of senescence-associated (SA) β-galactosidase (gal) staining in liver sections (200x). (A) 4 and 20 months old BL6 and TLR2-/- mice, (B) mice treated with the TLR2 inhibitor ortho-vanillin.BL6 – C57BL/6J, TLR2$^{-/-}$ – TLR2 Knockout.

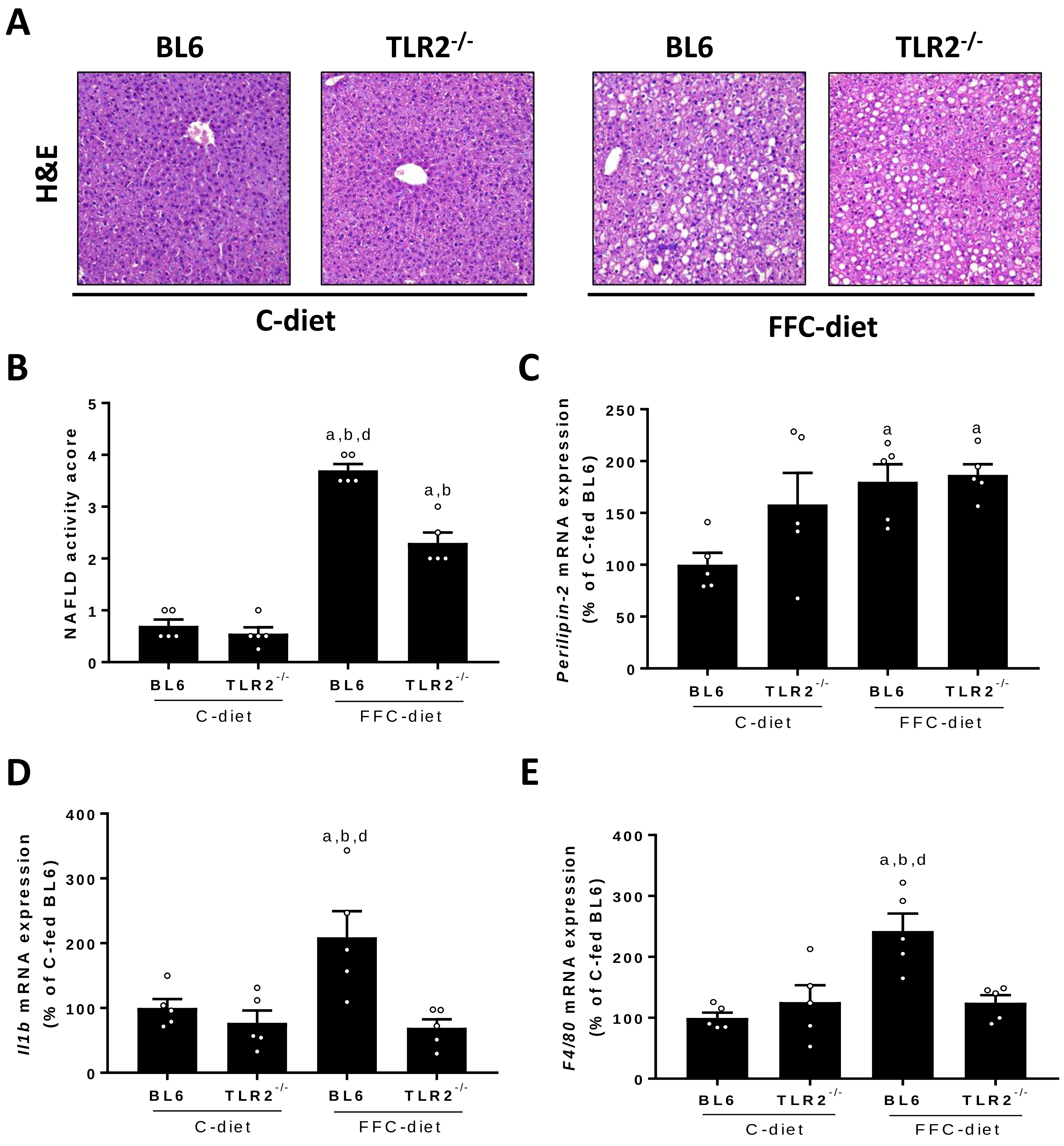

Supplemental Figure 3: Markers of liver damage in FFC-fed BL6 and TLR2$^{-/-}$ mice. (A) Representative pictures of hematoxylin and eosin (H&E) stained liver sections (200x), (B) NAFLD Activity Score, (C) *perilipin-2*, (D) *interleukin 1b* (*Il1b*) and (E) *F4/80* mRNA expression in liver of male BL6 and TLR2$^{-/-}$ mice fed a control (C) and fat-, fructose- and cholesterol- rich diet (FFC-diet). Data are presented as mean ± SEM, n=5, $^{a}p<0.05$ vs C-diet fed BL6 mice, $^{b}p<0.05$ vs C-diet fed TLR2$^{-/-}$ mice, $^{d}p<0.05$ vs FFC-diet fed TLR2$^{-/-}$ mice. *p*-values were determined by B-E: two-way ANOVA followed by Tukey´s Post Hoc test.

Supplemental Table 1. Nutrient composition of control and fat-, fructose- and cholesterol-rich diet (FFC-diet), both Ssniff, Germany

		C-diet	FFC-diet
**Crude protein, CP**	% wt/wt	17.4	16.0
**Crude fat, CL**	% wt/wt	5.1	11.8
**Crude fibre, CF**	% wt/wt	5.0	2.0
**Crude ash**	% wt/wt	4.1	4.2
**Corn Starch**	% wt/wt	34.6	5.0
**Sucrose**	% wt/wt	11.0	-
**Glucose**	% wt/wt	-	5.0
**Fructose**	% wt/wt	-	50.0
**Cholesterol**	% wt/wt	-	0.16
**L-Lysine**	% wt/wt	1.43	1.32
**L-Methionine + L-Cysteine**	% wt/wt	1.00	0.96
**L-Threonine**	% wt/wt	0.75	0.69
**Calcium**	% wt/wt	0.78	0.77
**Phosphorus**	% wt/wt	0.48	0.47
**Sodium**	% wt/wt	0.23	0.30
**Vitamin A (retinol acetate)**	IU/kg	15000	15000
**Vitamin D_3 (cholecalciferol)**	IU/kg	1500	1500
**Vitamin E (α-tocopherol acetate)**	mg/kg	150	150
**Fatty acids**			
**C4:0**	% wt/wt	-	0.44
**C6:0**	% wt/wt	-	0.29
**C8:0**	% wt/wt	-	0.16
**C10:0**	% wt/wt	-	0.35
**C12:0**	% wt/wt	-	0.40
**C14:0**	% wt/wt	0.02	1.23
**C16:0**	% wt/wt	0.57	3.10
**C18:0**	% wt/wt	0.18	1.14
**C18:1 (n-9)**	% wt/wt	1.28	2.58
**C18:2 (n-6)**	% wt/wt	2.65	0.21
**C18:3 (n-3)**	% wt/wt	0.29	0.06
**Metabolizable energy (ME)**	kcal/kg	3752	4254
**CP**	kcal%	19	15
**CL**	kcal%	12	25
**Carbohydrate, CHO**	kcal%	69	60

19

Supplemental Table 2. Primer list.

	Forward (5´-3´)	Reverse (5´-3´)
	human	
18S	GGG CCC GAA GCG TTT ACT TT	CGC CGG TCC AAG AAT TTC AC
p16	CCC ACC CCG CTT TCG TAG TT	CGC AAG AAA TGC CCA CAT GA
p21	GCC CCT CTG ACC TGC ACT GG	AGG GAC AGC AGC AGA GGG GA
	mouse	
18S	GTA ACC CGT TGA ACC CCA TT	CCA TCC AAT CGG TAG TAG CG
asma	CTG ACA GAG GCA CCA CTG AA	CAT CTC CAG AGT CCA GCA CA
F4/80	TGG CTG CCT CCC TGA CTT TC	CAA GAT CCC TGC CCT GCA CT
Il1b	TGG CTG TGG AGA AGC TGT GG	GTC CGA CAG CAC GAG GCT TT
Irf3	AAC CGG AAA GAA GTG TTG CG	GCA CCC AGA TGT ACG AAG TCC
Myd88	CCC TAG GGC AGA GGG GAA GA	ATG CCT GTG TGT GCA GAG GAG
p16	CCA AGA GCG GGG ACA TCA AG	AAG AAA AAG GCG GGC TGA GG
p21	GCC CCT CTG ACC TGC ACT GG	AGG GAC AGC AGC AGA GGG GA
Perilipin-2	GTGGGTGGAGTGGAAGAGAA	TGGCATGTAGTCTGGAGCTG
Tlr2	CTC CAC AAG CGG GAC TTC GT	GGC TCC AGC AAA ACA AGG A

Supplemental Table 3: Body weight and kcal intake in young, female BL6 and TLR2$^{-/-}$ mice treated with FFC-diet.

Parameter	Groups			
	C-diet		FFC-diet	
	BL6	TLR2$^{-/-}$	BL6	TLR2$^{-/-}$
**Body end weight (g)**	22.0 ± 0.4	22.2 ± 0.3	22.8 ± 0.7	22.7 ± 1.1
**kcal/mouse/day**	9.9 ± 0.1	9.7 ± 0.1	9.7 ± 0.1	9.6 ± 0.1
**Liver/body weight ratio (%)**	5.2 ± 0.2	4.8 ± 0.0	7.0 ± 0.2[a,b]	6.5 ± 0.2[a,b]

Data are presented as mean ± SEM, n=5, C – control, FFC – fat-, fructose- and cholesterol-rich, [a]p<0.05 vs C-diet fed BL6 mice, [b]p<0.05 vs C-diet fed TLR2$^{-/-}$ mice. *p*-values were determined by two-way ANOVA followed by Tukey´s Post Hoc test.
